# Analyzing Plant Gene Targeting Outcomes and Conversion Tracts with Nanopore Sequencing

**DOI:** 10.3390/ijms22189723

**Published:** 2021-09-08

**Authors:** Paul A. P. Atkins, Maria Elena S. Gamo, Daniel F. Voytas

**Affiliations:** 1 Department of Genetics, Cell Biology and Development, University of Minnesota, Minneapolis, MN 55455, USA; atkin265@umn.edu (P.A.P.A.); mgamo@umn.edu (M.E.S.G.); 2 Center for Precision Plant Genomics, University of Minnesota, St. Paul, MN 55108, USA; 3 Center for Genome Engineering, University of Minnesota, Minneapolis, MN 55455, USA

**Keywords:** genome engineering, homologous recombination, amplicon sequencing, bioinformatics

## Abstract

The high-throughput molecular analysis of gene targeting (GT) events is made technically challenging by the residual presetabce of donor molecules. Large donor molecules restrict primer placement, resulting in long amplicons that cannot be readily analyzed using standard NGS pipelines or qPCR-based approaches such as ddPCR. In plants, removal of excess donor is time and resource intensive, often requiring plant regeneration and weeks to months of effort. Here, we utilized Oxford Nanopore Amplicon Sequencing (ONAS) to bypass the limitations imposed by donor molecules with 1 kb of homology to the target and dissected GT outcomes at three loci in *Nicotiana benthamia* leaves. We developed a novel bioinformatic pipeline, Phased ANalysis of Genome Editing Amplicons (PANGEA), to reduce the effect of ONAS error on amplicon analysis and captured tens of thousands of somatic plant GT events. Additionally, PANGEA allowed us to collect thousands of GT conversion tracts 5 days after reagent delivery with no selection, revealing that most events utilized tracts less than 100 bp in length when incorporating an 18 bp or 3 bp insertion. These data demonstrate the usefulness of ONAS and PANGEA for plant GT analysis and provide a mechanistic basis for future plant GT optimization.

## 1. Introduction

The ability to efficiently modify any sequence within a plant genome would greatly accelerate basic and applied plant research. One method for making nucleotide-specific modifications is gene targeting (GT), wherein a locus is repaired by homologous recombination using a supplied ‘donor’ DNA as a template [1]. The efficiency of gene targeting has been found to be extremely variable within eukaryotes and to be uniformly enhanced by a targeted DNA double-strand break (DSB) at the modification site [2]. Due to its low efficiency in plants, the analysis of plant gene targeting outcomes has been largely restricted to phenotypic reporters that utilize a screen or selection to identify positive events. This has directed the application of gene targeting to a handful of endogenous and synthetic targets rather than genes underlying key agricultural traits, such as yield and disease resistance [3,4].

Genome editing outcomes are difficult to examine in the presence of donor molecules. Donors interfere with high-throughput molecular methods (e.g., Illumina sequencing and qPCR/ddPCR approaches) by limiting primer placement. Primers placed within the homology arms of a donor molecule will amplify both the target locus and donor molecules creating false positives, while primers placed outside the regions of homology will typically yield amplicons too large for analyses. While it is possible to remove the donor via long-term propagation or regeneration of edited cells, this process remains resource intensive and low throughput in plants [5].

Third-generation sequencing methods, PacBio and Oxford Nanopore Sequencing, make sequencing large amplicons possible. Both methods bypass donor-imposed amplicon size restrictions using long reads, readily accommodating several thousand basepair amplicons. PacBio sequencing has been previously used to analyze gene targeting events, but it requires a considerable capital investment and has inflexible run sizes compared to Oxford Nanopore Amplicon Sequencing (*ONAS*) [6,7]. For comparison, as of summer 2021, all hardware and reagents necessary for several ONAS runs (potentially hundreds of GT samples) can be acquired for under USD 5000. This includes an Oxford Nanopore MinION starter kit (a MinION sequencing device with several flow cells and library prep kits) and a desktop computer with a GPU capable of translating the MinION output into nucleotides in a timely fashion.

Here we demonstrate the utility of *ONAS* for the analysis of gene targeting events. To aid in our efforts, we developed a novel bioinformatic tool: Phased ANalysis of Genome Edited Amplicons (*PANGEA*). *PANGEA* accommodates *ONAS* error when identifying GT-positive reads, maintains phased information from individual reads for conversion tract analysis, and reduces the effect of sequencing error on estimates of targeted mutagenesis. This establishes an affordable, scalable, and high-throughput platform for the mechanistic analysis and optimization of gene targeting amenable to virtually any organism.

## 2. Results

**Figure 1:**
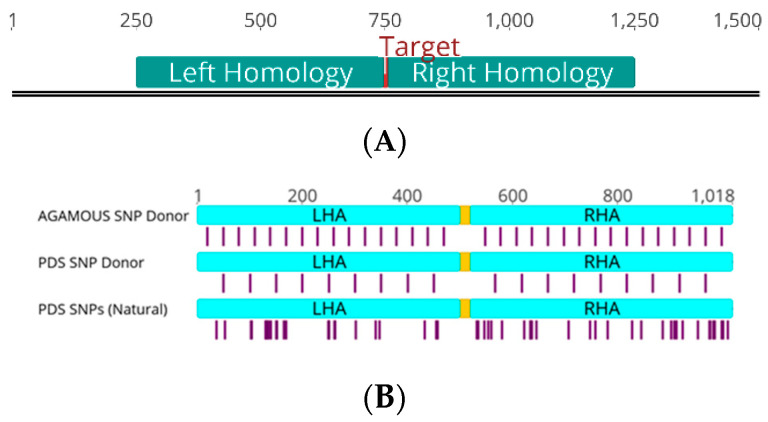
Generalized schema of target sites and donors. (**A**) Generalized genomic site for gene targeting using 500 bp homology arms. Left and Right Homology indicate regions with homology to donor arms. Blue arrows represent the primers that generate the smallest possible amplicon without spurious donor amplification. Target indicates the position of the targeted insertion and nuclease cut site. (**B**) Donor schema for divergent donors. *SNP* positions indicated by purple bars and yellow bars represent the targeted insertion. The targeted insertion for all donors is at the predicted Cas9 cut site, preventing cleavage of donors and HR repair products. Engineered *SNP* donors at *AGAMOUS* and *PDS* encode SNPs in 30bp and 50bp intervals, respectively. Divergent donors using natural variation between *PDS3.1* and *PDS3.2* have irregularly spaced *SNPs*. *LHA* and *RHA* indicate left homology arm and right homology arm, respectively.

T-DNA encoding genome editing reagents were delivered via leaf infiltration to transgenic *Nicotiana benthamiana* encoding a 35 S-driven Cas9 [8]. Targets for modification included two phytoene desaturase (*PDS*) paralogs (*PDS3.1* and *PDS3.2*), which share a common gRNA target site, and *AGAMOUS*. Each T-DNA delivered the gRNA and donor, which in some cases was on a geminiviral replicon (GVR). GVRs replicate inside plant cells and amplify the donor to high levels, resulting in elevated frequencies of GT [9]. Donor molecules with 500 bp homology arms direct either a 3 bp or 18 bp insertion to the predicted Cas9 cut site, disrupting the gRNA target in both the donor and the target site repaired by homologous recombination (Figure 1A,B).

Five days after leaf infiltration, DNA was extracted, and the target sites were amplified using barcoded, paralog-specific primers in triplicate to minimize PCR jackpotting (Table S1). The resulting amplicons were pooled, a library was prepared using a ligation-based kit (LSK109) and the library was sequenced using ONAS. The resulting fast5 files were basecalled using Guppy, demultiplexed using minibar, and aligned to the appropriate reference using minimap2 [10,11,12].

To leverage ONAS’s advantages, we developed a bioinformatic pipeline, PANGEA, to analyze gene targeting outcomes from nanopore reads that accommodates its primary shortcoming: a high error rate [13]. For each read within the resulting alignment file, PANGEA parses CIGAR strings for insertions and deletions within 4 bp of the predicted cut site (target window) and, if applicable, examines all positions for SNPs matching those in the donor’s homology arms (Figure S1).

Using PANGEA, the percentage of reads having at least 1 variant in the target window varied by target and treatment, with non-nuclease samples at *AGAMOUS* having less than half the level of non-nuclease *PDS* samples (mean of 7.3%, 20.2%, and 17.1% at *AGAMOUS*, *PDS3.1*, and *PDS3.2*, respectively, Figure S2). Nuclease-treated samples had a much broader distribution and significantly higher frequency of variants, presumably due to the variable activity of the nuclease in each sample (Figure S2). We sought to harness the consistency of *ONAS* error to reduce its contribution to estimates of targeted mutagenesis using a subtraction strategy; variants found within a non-nuclease control sample were subtracted from each treated sample on a variant-specific basis (Figures S3 and S4). After subtraction, the sum of all positive variant frequencies above 0.5% was used as the adjusted targeted mutagenesis frequency.

To identify gene targeting modifications in *ONAS* amplicon sequences, *PANGEA* performs a ‘fuzzy’ search. Each read is examined for variants matching the expected donor modifications based on an alignment of the donor to the target reference. Insertions within the target window whose sequence are within a Levenshtein distance (Ld) of 1/3 the size of the modification (Ld ≤ 6 for an 18 bp insertions and Ld ≤ 1 for 3bp insertions) were counted as GT-positive reads (Figure S1A). This method was found to produce extremely small GT counts (<0.06%) in samples lacking either a nuclease or a donor when searching for an 18bp insertion (Figures S5 and S6).

**Figure 2:**
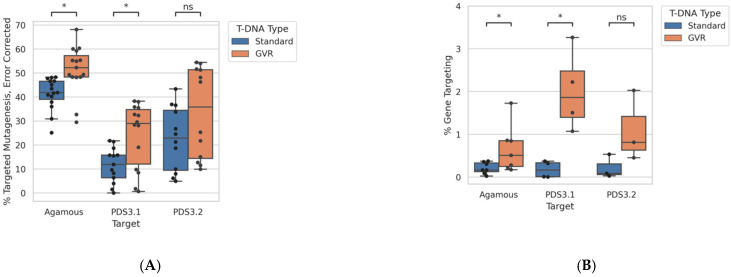

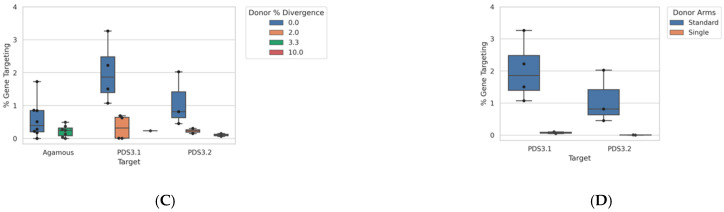
Efficiency of genome editing as assessed by *ONAS* and *PANGEA*. (**A**) Corrected percent targeted mutagenesis at three loci when delivered a nuclease with and without a GVR. (**B**) Percent gene targeting at three loci when delivered nuclease and donor with 500 bp homology arms encoding a 18bp insertion with and without a GVR. (**C**) Percent gene targeting using donors of various divergence; 2.0% and 3.3% diverged donors use engineered SNPs at a consistent interval (50 bp and 33 bp, respectively) while the 10.0% diverged donors use the natural variance between the two *PDS* loci. All samples were delivered with a nuclease and GVR. Values at *PDS3.1* correspond to 0.0, 1.0, and 10.0, respectively. (**D**) Percent gene targeting at *PDS3.1* and *PDS3.2* when using two 500 bp donor arms or a single 500 bp donor arm. All samples delivered with a nuclease and GVR. * Indicates a *p*-value < 0.05 from a Mann–Whitney test. ns—no statistical significance.

Using *ONAS* and *PANGEA*, genome editing outcomes were assessed at *AGAMOUS*, *PDS3.1*, and *PDS3.2*. Targeted mutagenesis efficiency at the *AGAMOUS* and *PDS3.1* loci were significantly increased by the GVR (Figure 2A). Background errors at *AGAMOUS* and both *PDS* loci were not equal, with *PDS* targets having higher ONAS background in untreated samples (Figure S7). Gene targeting frequencies were also significantly increased by the presence of the GVR at two of three loci (Figure 2B). *PDS3.1* and *PDS3.2* were exclusively delivered donors encoding 18 bp insertions, while *AGAMOUS* was delivered donors with either a 18 bp or 3 bp insertion (Figure 2B). No detectable difference in GT efficiency was found at *AGAMOUS* when delivered donors encoding 3bp and 18 bp (Figure S8).

Next, we examined *GT* outcomes using donors with imperfect homology. Imperfect homology took the form of regularly interspersed SNPs (every 30 bp or 50 bp for *AGAMOUS* and *PDS*, respectively) or the naturally occurring 10% sequence divergence between *PDS3.1* and *PDS3.2* (Figure 1B and Figure 2C). In the case of natural sequence variation, the PDS3.1 donor was used to repair *PDS3.2*, and vice versa. Donors with imperfect homology performed poorly even when delivered with a GVR and nuclease, with fold reductions in *GT* frequency ranging from 0.38 at *AGAMOUS* and 0.07 at *PDS3.2* when delivered donors diverged by 3.33% (regular SNPs every 30 bp) or 10% (natural variation between *PDS* paralogs), respectively (Figure 2C). Gene targeting frequencies were also drastically reduced when donors used a single homology arm instead of two (Figure 2D). These expected differences when using a divergent donor or a single homology arm were unable to be observed when the GVR was not used, suggesting non-GVR treatments with standard donors and all treatments using imperfect homology donors may be outside the linear dynamic range of *ONAS* (Figures S9 and S10). 

To monitor conversion tracts copied from imperfect donors, *PANGEA* catalogs all SNPs between the donor and reference. Within each read, donor SNPs are marked as present or absent (Figures S11 and S12). *ONAS* error in SNPs was partially mitigated by ignoring any *SNP* external to 3 consecutive WT SNPs, removing virtually all background outside the 3 innermost *SNPs* (Figure S1B). To correct donor SNPs from being removed within conversion tracts as *ONAS* error, *WT* positions found within a putative conversion tract (as determined by the previous step) were ‘filled in’ (Figure S1B).

**Figure 3:**
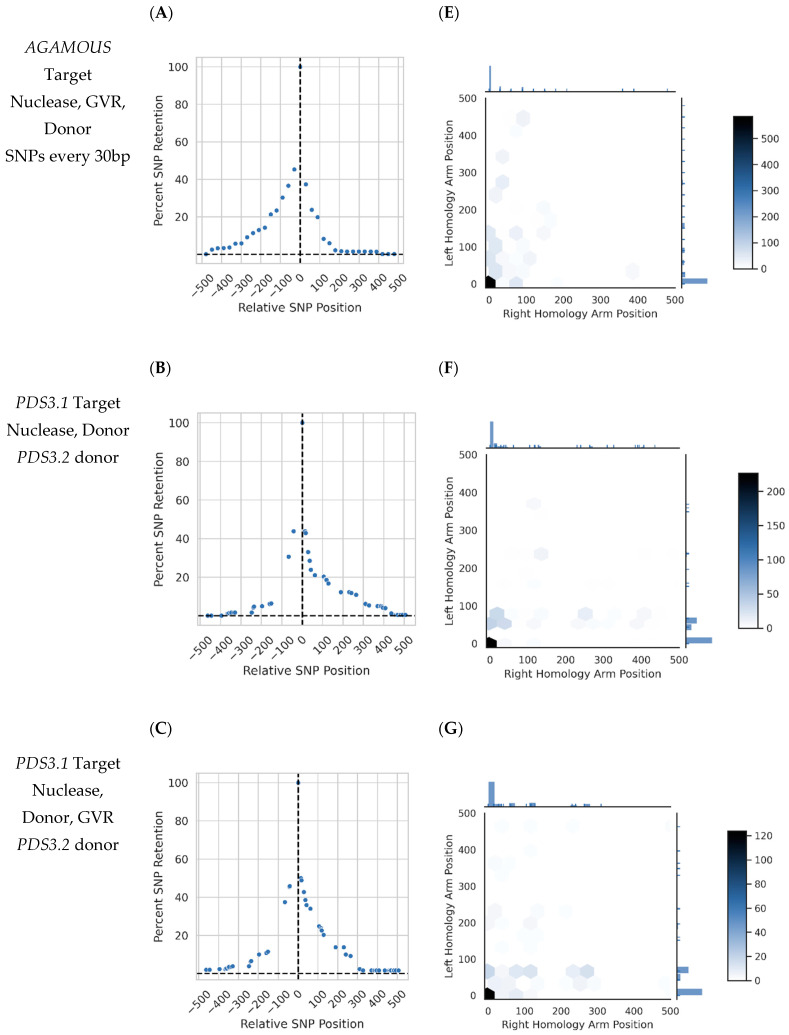

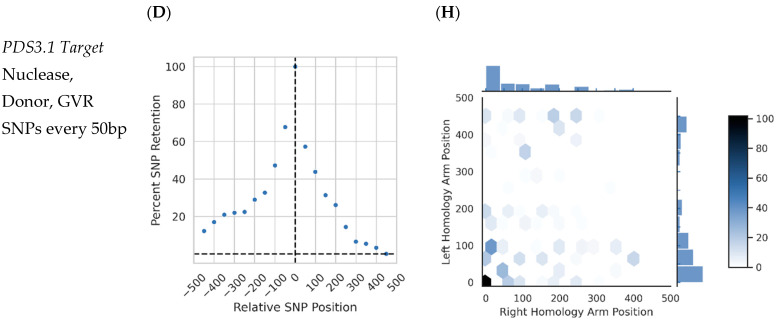
Conversion tracts collected from four gene targeting samples (a representative subset) plotted using two approaches. (**A**–**D**) Conversion tract diagram showing the percentage of GT reads containing individual donor SNPs. All donor SNPs are represented on the plot and their position relative to the GT insertion is noted on the X axis, with 0 being the GT insertion itself. SNP patterns were ‘smoothed’ to accommodate *ONAS* error (see text and Figure S1B). (**E**,**F**) Hexbin plots showing conversion tract patterns in 2 dimensions. Right and Left Homology Arm Position indicate the outermost *SNP* copied into the target locus from the respective donor arm (right or left). Color intensity indicates the count of events at each position. Axis histograms show relative frequency of conversion tract size for individual arms. (**A**,**E**) *AGAMOUS* targeted using a nuclease, GVR, and donor encoding a 3bp insertion with *SNPs* every 30 bases. (**B**,**F**) *PDS3.1* targeted using a nuclease and donor encoding an 18bp insertion with perfect homology to *PDS3.2*, an approximately 10% diverged template with an uneven *SNP* distribution. (**C**,**G**) As in panels B and F, but with the addition of the GVR. (**D**,**H**) *PDS3.1* targeted using a nuclease, GVR, and engineered donor with *SNPs* every 50 bp.

*PANGEA* was used to assess the distribution of conversion tracts in gene targeting events. Strikingly, most conversion tracts at *AGAMOUS* and *PDS3.1* were under 100 bp (Figure 3A–D). Only a single treatment contained more than 100 GT events using diverged donors at *PDS3.2*, limiting our ability to interpret tract patterns across treatments at *PDS3.2* (Table S5). Short conversion tracts were found in non-SNP controls, which is consistent with *ONAS* error and the error reduction strategy not functioning at the 3 most internal *SNPs* (Figures S11 and S13). The same SNP conversion tract information was plotted using heatmaps to preserve the phased information present in the long reads, revealing the two-dimensional distribution of conversion tracts in somatic plant gene targeting events (Figure 3E–H).

## 3. Discussion

Here we analyzed somatic GT outcomes in *N. benthamiana* leaf tissue with ONAS by developing PANGEA, a bioinformatic approach that reduces the effects of sequencing error and preserves phased information from long reads to enable conversion tract analysis. We found that, unlike outcomes of targeted mutagenesis, gene targeting events are readily distinguishable from sequencing error with minimal accommodation (Figure 2A,B) and Figure S1A. For the GT reagents assessed here, nuclease-only (non-donor) samples were scored as less than 0.06% gene targeting, making ONAS an ideal approach for analyzing GT outcomes in the presence of residual donor molecules (Figure S4).

GT was readily detectable in samples delivered a donor with 500 bp homology arms and a GVR. The GVR significantly increased GT and targeted mutagenesis frequencies at two of three targets (Figure 2A,B). The observed GT frequencies in non-GVR and/or imperfect donor treatments suggest these events are below the linear dynamic range of the assay, making metrics comparing GVR to non-GVR treatments uninformative. Precise quantification of these rare events with long amplicons may be possible in future work by the use of unique molecular identifiers [14].

ONAS and PANGEA facilitated the collection of thousands of GT homology tracts across several treatments (Figure 3 and Figure S12). Minimal SNP noise control measures restricted background conversion tracts to the three most internal SNPs in each arm (Figures S1A and S10–S12). Hexbins present information masked by standard conversion tract representations, in particular the unidirectionality or bidirectionality of conversion tracts that indicate synthesis-dependent strand annealing (SDSA) repair or a double Holliday junction (dHJ) intermediate, respectively (Figure 3E–H) [15].

Our approach also revealed that many samples had a general bias in SNP incorporation and that conversion tract patterns may be altered by the GT reagents or target (Figure 3E–H). This may be the effect of the uneven divergence of *PDS3.1* and *PDS3.2*’s homology arms; the more SNP-dense right arm may have been preferentially incorporated due to invasion being more likely to occur within the left arm (Figure 1B and Figure 3F). It should be noted that the 4th innermost SNP in the *PDS* natural variant donor is 153bp away from the targeted insertion, making the 3 SNPs inside that range possible background based on SNP patterns in non-GT reads (Figure S12). Previous studies in mammals and yeast have demonstrated that donor divergence is not expected to mechanistically alter outcomes, only the available sites for homologous recombination initiation [16,17,18].

‘Coldspots’ in the conversion tract patterns are an additional source of information, as many potential outcomes are never captured in thousands of events across dozens of samples. The near-complete lack of events incorporating SNPs over 300 bp and the dominance of zero SNP incorporation in all samples, even in the presence of background at the 3 most internal SNPs, is highly conspicuous (Figure 3 and Figure S12). These together suggest that *N. benthamiana* may be limited in its ability to incorporate large fragments of DNA in somatic tissue or that conversion tract length can vary drastically for both sub-pathways of homologous recombination (dHJ and SDSA) depending on tissue and cell state.

The extremely short conversion tracts found here differ from previous observations in humans and plants [16,19,20,21]. Differences in cell state between studies (here non-dividing cells and non-selected compared to actively dividing cells with GT-specific selection) may at least partially explain conversion tract length differences, as homologous recombination is tightly regulated by the cell cycle [22]. Additionally, the short time frame (five days) may have resulted in the patterns captured here, as homologous recombination can occur over days, although large insertions have been detected within this timeframe using phenotypic assays in *Nicotiana tabacum* [9,23]. Because the GT conversion tract patterns observed here are far from ideal for making many desirable genome modifications, efforts to determine how GT is mechanistically occurring may be a reasonable troubleshooting step in tissues or organisms where GT reagents perform poorly. The combination of ONAS and PANGEA makes such experiments in many systems much more feasible to perform.

We provide data demonstrating a high-throughput nanopore-based pipeline for assessing gene targeting outcomes and mechanistic analysis, bypassing donor-imposed limitations. PANGEA readily accommodates ONAS error when searching for GT events and when examining conversion tracts, making direct and high-throughput mechanistic analysis of GT outcomes within virtually any tissue possible. Our analysis sheds light onto the nature of somatic plant gene targeting events, particularly the high usage of extremely short conversion tracts, and establishes a platform for guiding future GT optimization with mechanistic insights.

## 4. Materials and Methods

### 4.1. Vector Construction

T-DNA plasmids were constructed using a previously published Golden Gate plant genome engineering toolkit [24]. Synthesized fragments and amplicons used to create final T-DNA vectors are listed in Tables S2–S4. The *N. benthamiana PDS* and *AGAMOUS* gRNAs were described previously [25]. *AGAMOUS*, *PDS3.1* and *PDS3.2* donor molecules (with and without SNPs) were synthesized by Twist Bioscience. *PDS3.1*, *PDS3.2*, and *AGAMOUS* donors were designed using the *N. benthamiana* genome v1.0.1 at www.solgenomics.net, accessed on 7 January 2021 (Niben101Scf01283g02002.1, Niben101Scf14708g00023.1, and Niben101Scf12205g00011.1, respectively).

### 4.2. Plant Material

*N. benthamiana* encoding a transgene expressing Cas9 under the 35S promoter has been previously described [8]. These plants were grown at 24C and 60% humidity, 16h/8h day/night cycle in a Conviron growth chamber. Plants were selected for infiltration after 4–5 weeks of growth.

### 4.3. Leaf Infiltration and DNA Isolation

The GV3101 Agrobacterium strain was transformed via the freeze-thaw method and delivered to true leaves via leaf infiltration as previously described [9]. DNA was extracted from infiltrated plants 5 days post infiltration using a plate-based CTAB method [26].

### 4.4. PCR Amplification of Target Sites

Primers and barcodes used for PCR amplification of *AGAMOUS*, *PDS3.1* and *PDS3.2* are described in Table S1. Amplifications were performed using PrimeStar GXL Polymerase using the manufacturer’s guidelines. 3x 25ul PCRs were performed for each sample at both *PDS3.1* and *PDS3.2*. Approximately 2ul of each sample was examined on an agarose gel to verify successful amplification. The 3 PCR replicates were then pooled and purified using a Qiagen PCR Purification Kit. DNA concentrations of the purified amplicons were determined using a NanoDrop and equimolarly pooled. Up to 36 samples from the same target were multiplexed in a single library for sequencing.

### 4.5. Nanopore Library Preparation and Sequencing

The SQK-LSK109 Oxford Nanopore Ligation Sequencing Kit (Oxford Nanopore Technologies, Oxford, UK) was used for library preparation, which was performed according to the manufacturer’s specifications using the Short Fragment Buffer (SFB) protocol variant. The completed library was sequenced using a R9.4.1 MinION flow cell on a MinION device (Oxford Nanopore Technologies, Oxford, UK) according to the manufacturer’s specifications.

### 4.6. Basecalling, Demultiplexing, and Additional Bioinformatics

Fast5 files were converted to nucleotide base calls using Oxford Nanopore’s Guppy Basecalling Software, version 3.3.3 + fa743a6 using an RTX2080 TI and Ubuntu 18.04 [12]. The resulting fastq files were demultiplexed using Minibar [11]. Additional bioinformatics were performed using Python 3.7 (Python Software Foundation, www.python.com, accessed on 1 December 2019) and graphed using Seaborn (https://seaborn.pydata.org/, accessed on 11 December 2019) and pandas (https://pandas.pydata.org/, accessed on 1 December 2019). A summary of PANGEA output for all individual samples can be found in Table S5. The PANGEA python script and executable used for the analysis of genome editing outcomes in nanopore reads can be found at https://github.com/papatkins/PANGEA, accessed on 1 December 2019.

## Data Availability

All code and outputs generated are available at github.com/papatkins/PANGEA. Fastq files are available upon request.

## Supplementary Materials

The following are available online at https://www.mdpi.com/article/10.3390/ijms22189723/s1.
