# Supplementary material for: Analyzing Plant Gene Targeting Outcomes and Conversion Tracts with Nanopore Sequencing
Source: Int J Mol Sci. 2021 Sep 8;22(18):9723. doi: 10.3390/ijms22189723 (PMC8467259; doi:10.3390/ijms22189723)
Supplement: Supplementary file 1 [file ijms-22-09723-s001.zip › atkins_GT_IJMS_supplemental_figs.pdf]

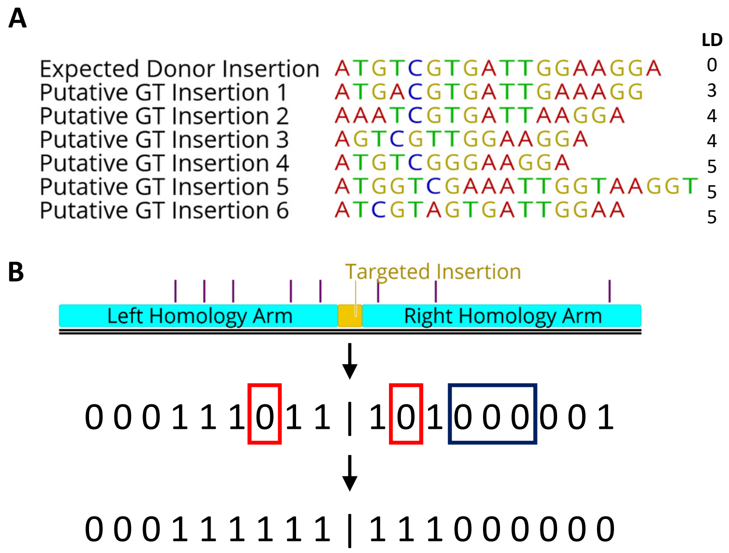

Figure S1. Error mitigation strategies when searching for GT events and conversion tracts in Nanopore amplicons. **A)** Example putative GT modifications compared to the expected donor insertion. Insertions scored as GT events due to the Levenshtein Distance (LD) between the insert and the expected modification beingsx less than or equal to 1/3 the expected insert size (18bp insertion, LD of 6). **B)** Example amplicon from a putative GT-positive read as analyzed by PANGEA. A subset of all potential donor SNPs are found within the homology arms. SNPs are extracted and converted to a binary format. Spurious SNP calls are reduced using two methods: first, the most interior 3 consecutive SNPS are identified and all exterior SNPs are ignored (blue box), Second, non-SNPs within putative conversion tracts are converted to SNPs (red boxes).

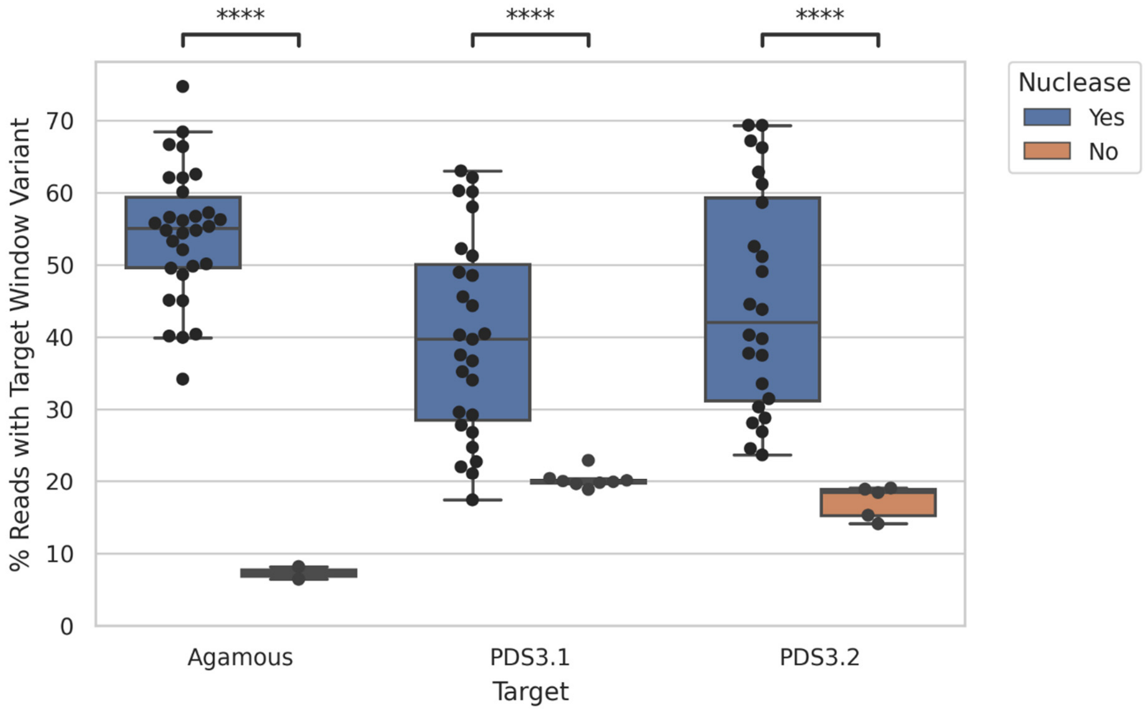

Figure S2. Nuclease-induced mutations are readily detectable above nanopore error. Percentage of reads from nuclease and non-nuclease treatments with a variant (non-reference sequence) in the nuclease target window (within 4bp of predicted cut site) at three targets. **** indicates p-value < $1x10^{-4}$ from Welch's t-test with Bonferroni correction (*AGAMOUS* = $5.1x10^{-13}$, *PDS3.1* = $2.0x10^{-7}$, *PDS3.2* = $1.8x10^{-8}$)

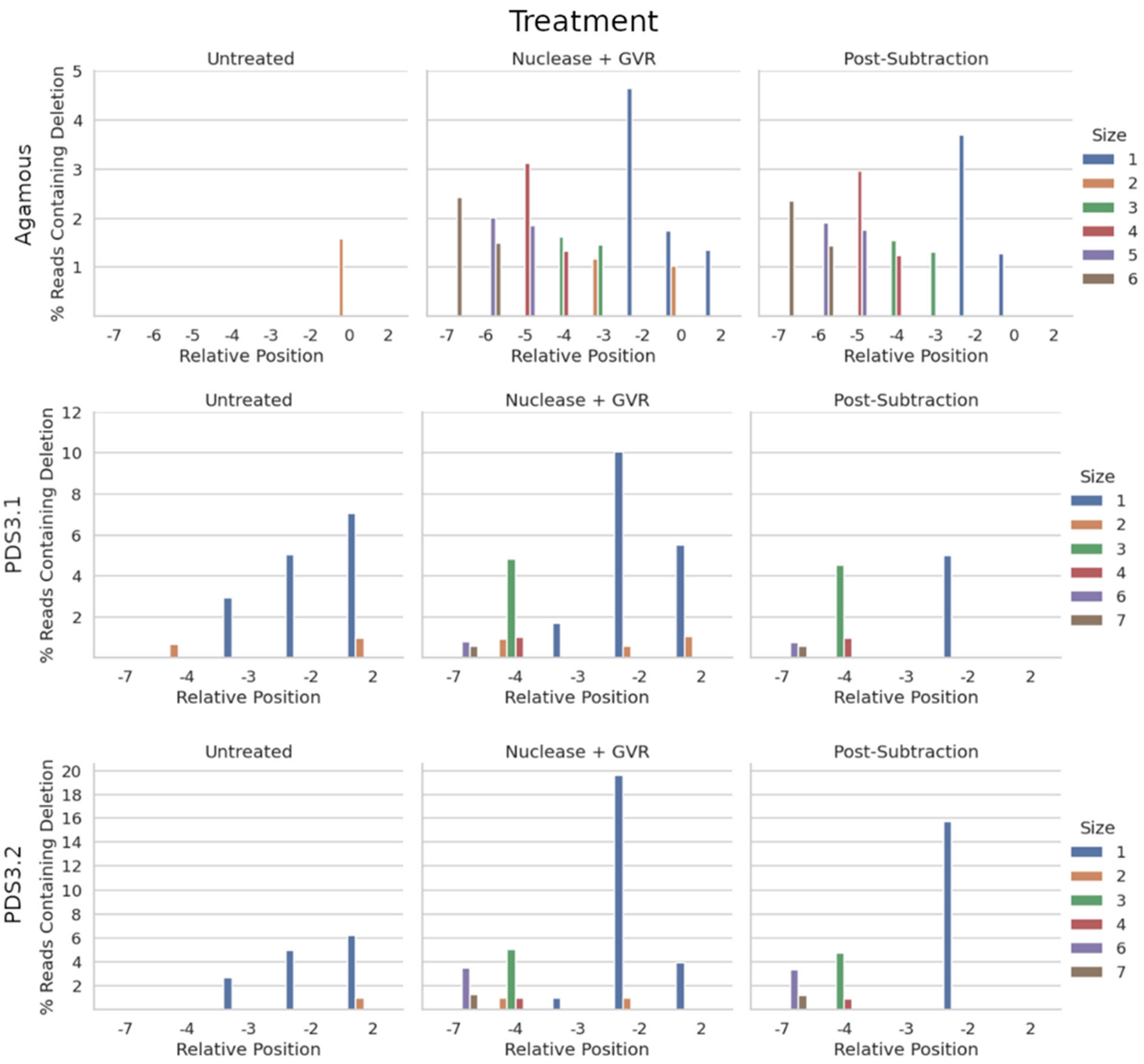

Figure S3. Deletions found in PANGEA-analyzed amplicons in ‘Untreated’ and ‘Nuclease and GVR’ samples and the outcome of the subtraction error reduction strategy at three targets from representative samples. Relative position is the distance from the predicted nuclease cut site. Numbers outside the expected target window (e.g., position -7 when target window is 4) are due to the deletion including the target window (e.g., 6bp deletion at position -7 deletes bases within the target window). Size indicates the size of the deletion at the given position. Post-subtraction values were calculated by subtracting the ‘Untreated’ value from the ‘Nuclease and GVR’ on a deletion-specific basis. Variants below 0.5% were ignored.

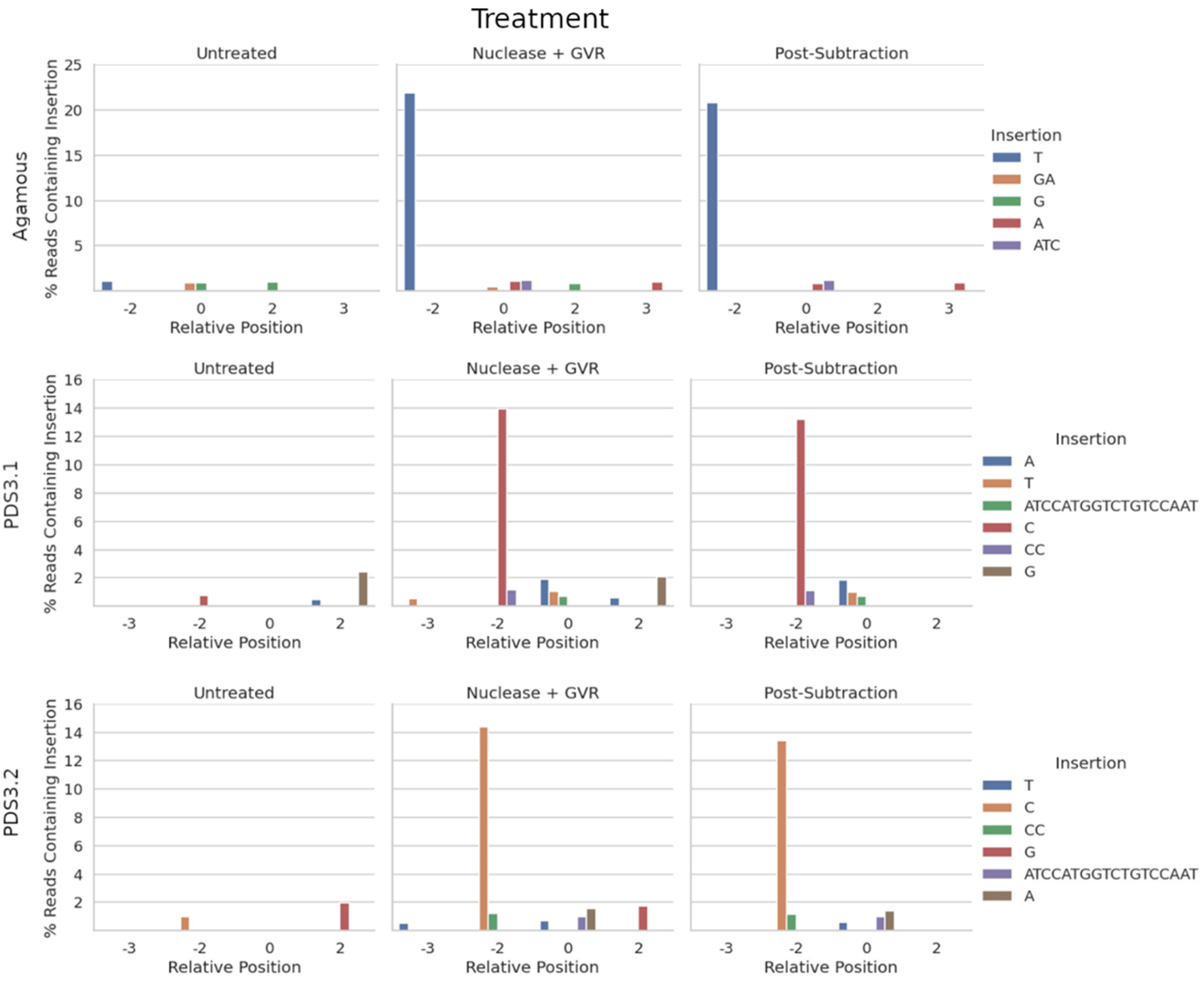

Figure S4. Insertions found in PANGEA-analyzed amplicons in 'Untreated' and 'Nuclease and GVR' samples and the outcome of the subtraction error reduction strategy at three targets from representative samples. Relative position is the distance from the predicted nuclease cut site. Insertion (legend key) indicates the exact nucleotide sequence of the insertion at the given position. Large insertions are the expected gene targeting insertion. A single read may contribute multiple distinct insertions. Post-subtraction values were calculated by subtracting the 'Untreated' value from the 'Nuclease and GVR' on an insertion-specific basis. Insertions below 0.5% were ignored.

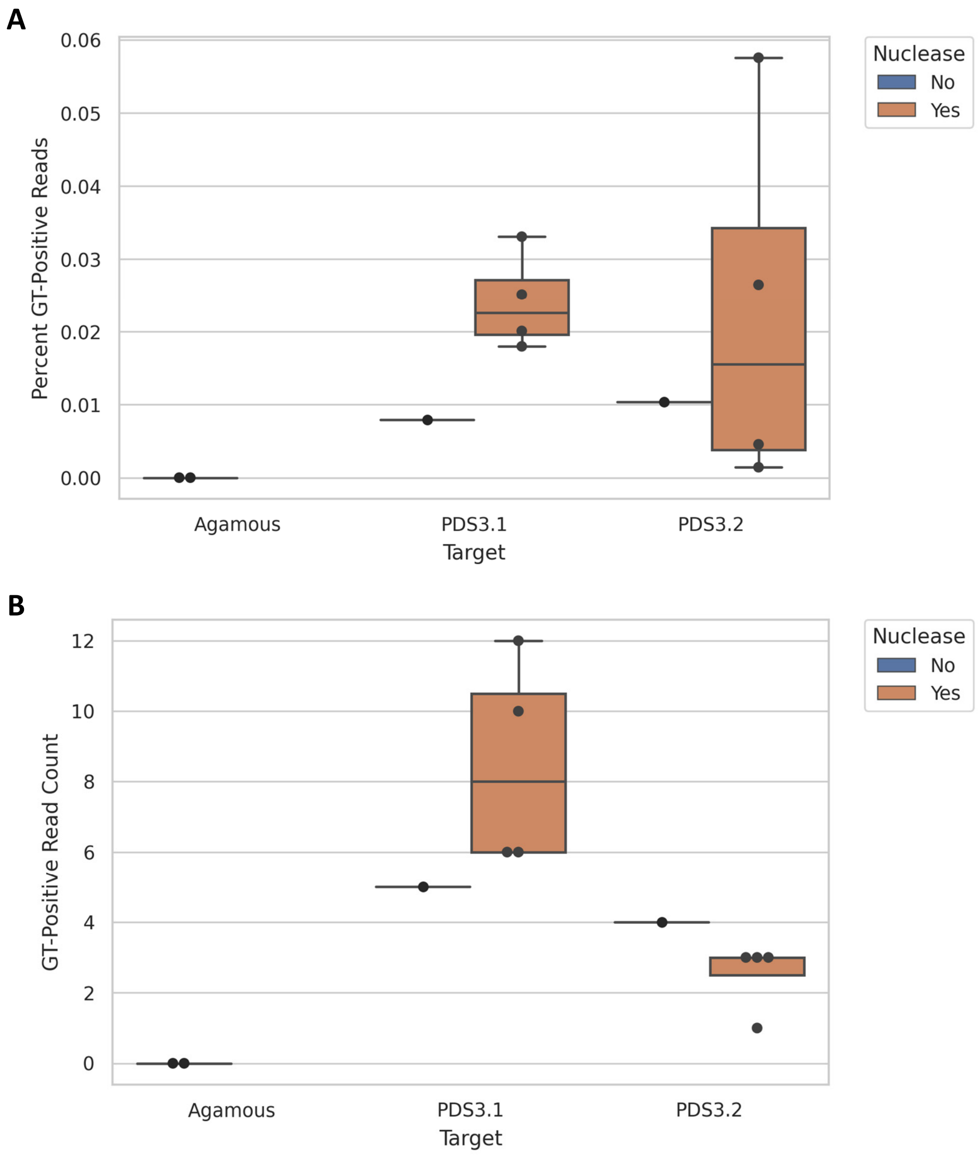

Figure S5. Use of 1/3 Levenshtein distance search produces very few false-positive GT events. Background levels of gene targeting found using PANGEA when searching for insertions within 6 Levenshtein distance of the expected 18bp GT insertion at three targets in non-donor samples (with and without nuclease). **A)** Percent GT-positive reads found at three targets. **B)** GT-positive read count at three targets.

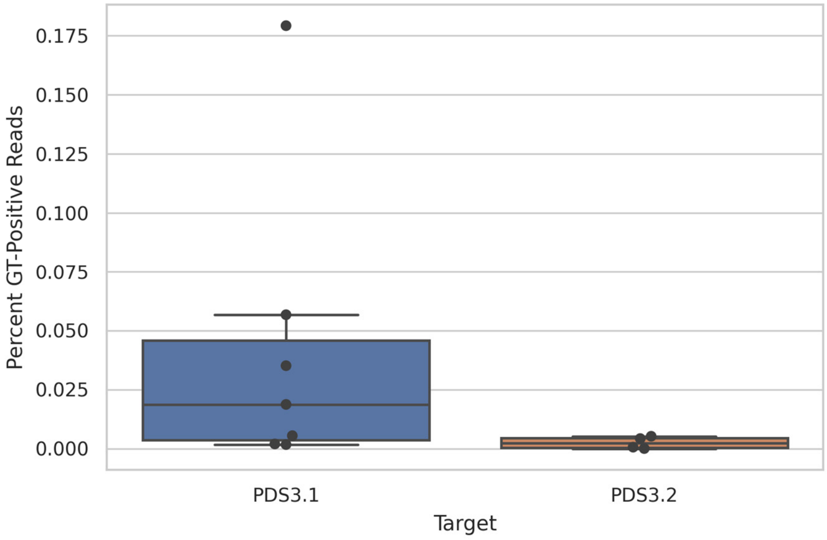

Figure S6. PDS treatments lacking a nuclease may be outside the limit of detection**.** Levels of gene targeting found using PANGEA when searching for insertions within 6 Levenshtein distance of the expected 18bp GT insertion at both *PDS* loci in samples delivered a donor molecule with or without a GVR (data combined) and no nuclease. No significant difference was found between donor molecule alone and donor molecule plus GVR at *PDS3.1* or *PDS3.2* (data not shown).

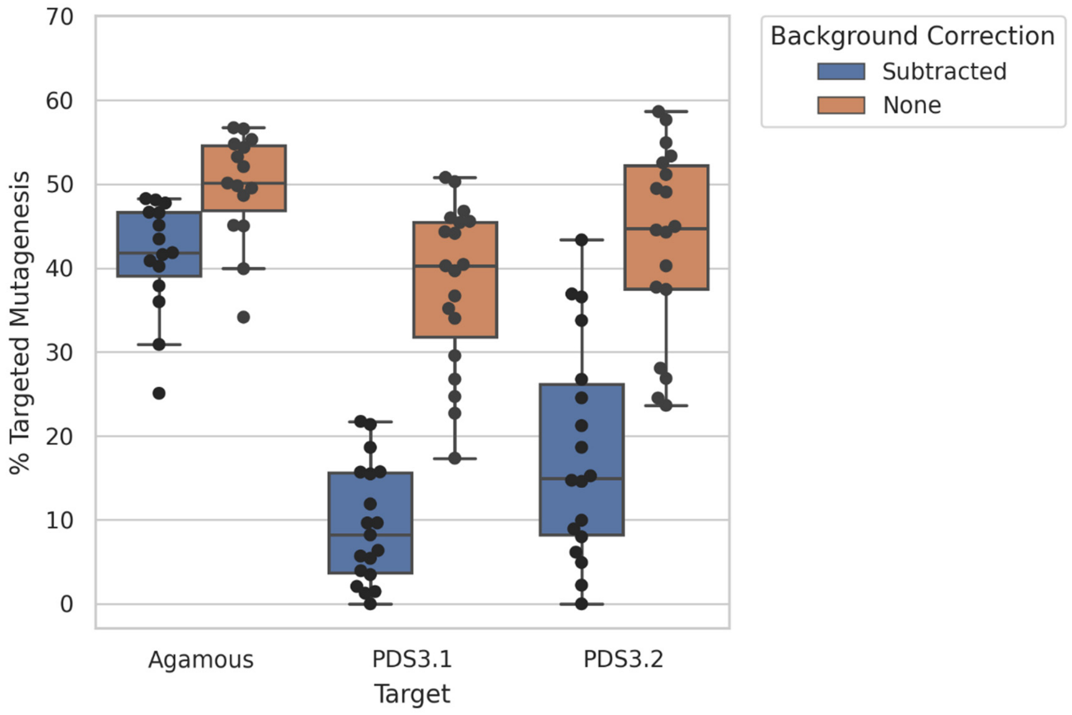

Figure S7. Effect of nanopore error background subtraction on estimates of targeted mutagenesis at three loci. Percent of reads with a sequence variant within the target window at three loci before and after background correction in nuclease treated samples. Variants found in the control sample at each locus were subtracted from the experimental samples on a variant-specific basis (see Supplemental Figures 2-3).

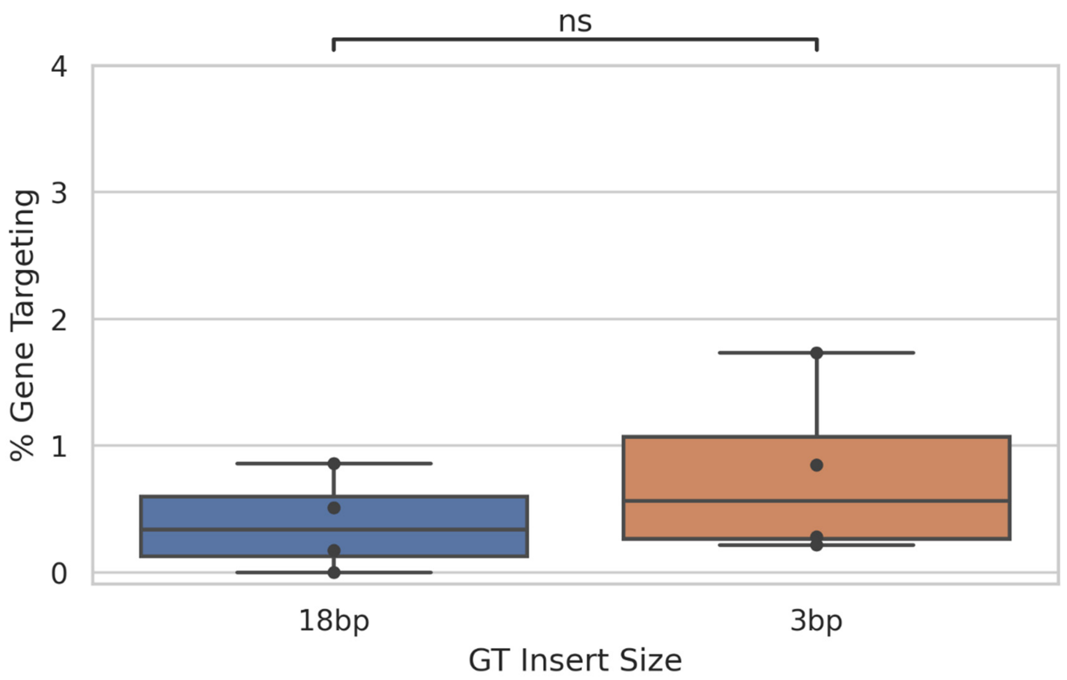

Figure S8. 18bp and 3bp insertions at *AGAMOUS* perform similarly. Comparison of gene targeting frequencies at the *AGAMOUS* locus using donors encoding insertions of different sizes delivered with a nuclease and GVR. No statistical difference found using Mann-Whitney test.

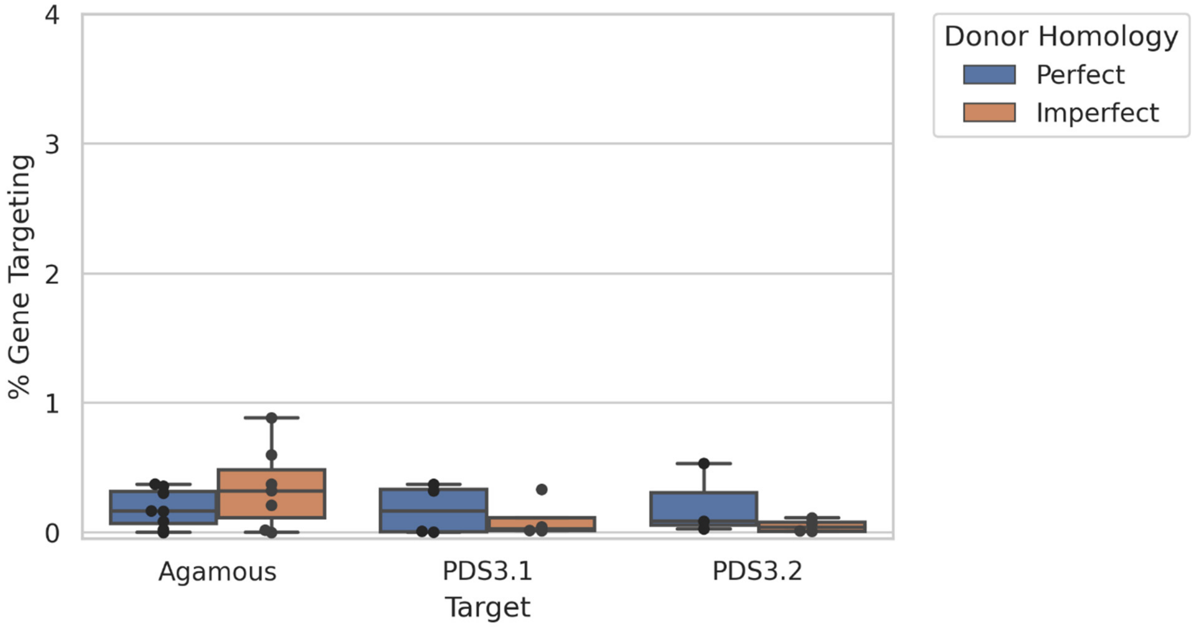

Figure S9. Non-GVR treatments are likely outside the limit of detection. Comparison of gene targeting efficiencies at three loci using donors of imperfect or perfect homology when delivered with a nuclease and *no GVR*.

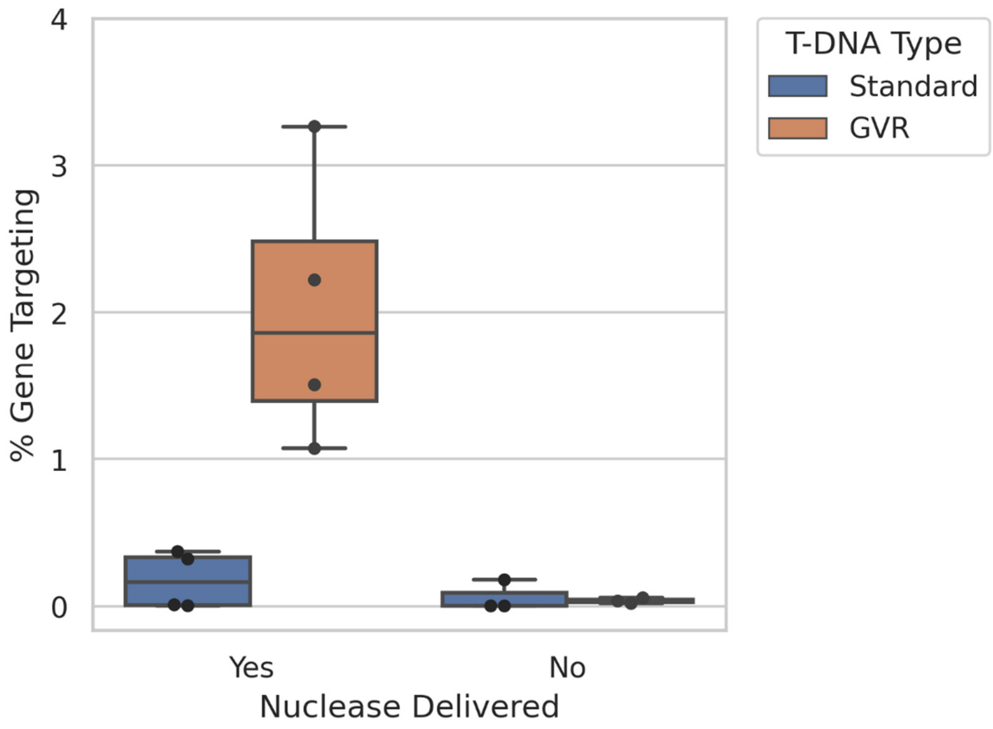

Figure S10. GVR results in readily detectable GT events. Gene targeting efficiency at *PDS3.1* when using combinations of nuclease and GVR. Readily quantifiable events are only detectable when both the GVR and nuclease are delivered.

Pattern	Count
0000000000000000000000000000000000000000000000000000000	67526
0000000000000000000000000001000000000000000000000000000	3403
0000000000000000000000000000000001000000000000000000000	2321
0000000000000000000000000000000000000000000100000000000	1872
0000000000000000000000000000000000000000000000000001000	1488
0000000000000000000000000000000000000000000000100000000	1180
0000000000000000000000000000010000000000000000000000000	1115
0000000000000000000000000000000100000000000000000000000	938
0000000000000000010000000000000000000000000000000000000	853
0000000000000000000000000000100000000000000000000000000	768
0000000000000000000000000000000000000100000000000000000	762
0100000000000000000000000000000000000000000000000000000	710
0000000000000000000000000000000000000000000010000000000	635
0000000000000000000000000000000000001000000000000000000	632
0000000000000000000000000000000000000001000000000000000	573
0000000100000000000000000000000000000000000000000000000	517
0000000001000000000000000000000000000000000000000000000	489
0000000000000000000010000000000000000000000000000000000	464
0000000000100000000000000000000000000000000000000000000	437
0000000000000000000000001000000000000000000000000000000	411

Figure S11. Example partial SNP conversion tract output from a negative control prior to noise reduction. A *PDS3.1* negative control sample was searched for donor SNPs corresponding to SNPs present in the *PDS3.*2 donor arms. Output prior to SNP noise reduction. Each position represents a potential donor SNP, in this case from the *PDS3.2* donor. ‘1’ indicates the presence of the donor SNP within the read and ‘0’ a non-donor SNP. ONAS error results in spurious donor SNPs; downstream processing ignores all SNPs that have 3 consecutive non-donor SNPs. For this sample, all donor SNPs except those at the 3 innermost positions on either side of the GT insertion will be reverted due to 3 consecutive internal non-SNP positions. In this example only the SNP in bottommost pattern would not be removed, resulting in an extremely low rate of false positives. Blue line indicates the relative position of the expected GT insertion.

Pattern	Count
0000000000000000000000000000000000000000000000000000000	20
0000000000000000000001111100000000000000000000000000000	9
0000000000000000000000001111110000000000000000000000000	6
0000000000000000000001111111111111111110000000000000000	5
0000000000000000000000000000100000000000000000000000000	5
0000000000000000000000111100000000000000000000000000000	3
0000000000000000000000001100000000000000000000000000000	3
0000000001000000000001111111110000000000000000000000000	2
0000000000000000001110110000000000000000000000000000000	2
0000000000000000000001111111110111100000000000000000000	2
0100000000000000000000000000100000000000000000000000000	2
0000000000000000000000000001100000000000000000000000000	2
0000000000000000000000000001000000000000000000000000000	2
0000000000000000000001111111111111000000000000000000000	2
0000000000000000000000000000000100000000000000000000000	2
0000000000000000000000001111100000000000000000000000000	2
0000000000000000000001111101100110000000000000000000000	1
0000000000000000000111111110101111100000000000000000000	1
0000000000000000001111100010000000000000000000000000000	1
1111111011111000011111111101111111100000000000000000000	1

Figure S12. Example raw partial SNP conversion tract output from GT-positive reads prior to noise reduction. A single *PDS3.1* sample was delivered a *PDS3.2* (10% divergent) donor, nuclease, and GVR and putative GT-positive amplicons were searched for SNPs corresponding those found in the *PDS3.2* donor. The most common conversion tract pattern found incorporates no SNPs (20 reads). Noise reduction will eliminate most outer SNPs and shorten the left conversion tract of the bottommost event (3+ consecutive non-SNPs results in all outer positions being ignored). Conversion tracts missing one or two neighboring internal SNPs will be ‘filled in’. Blue line indicates the relative position of the expected GT insertion.

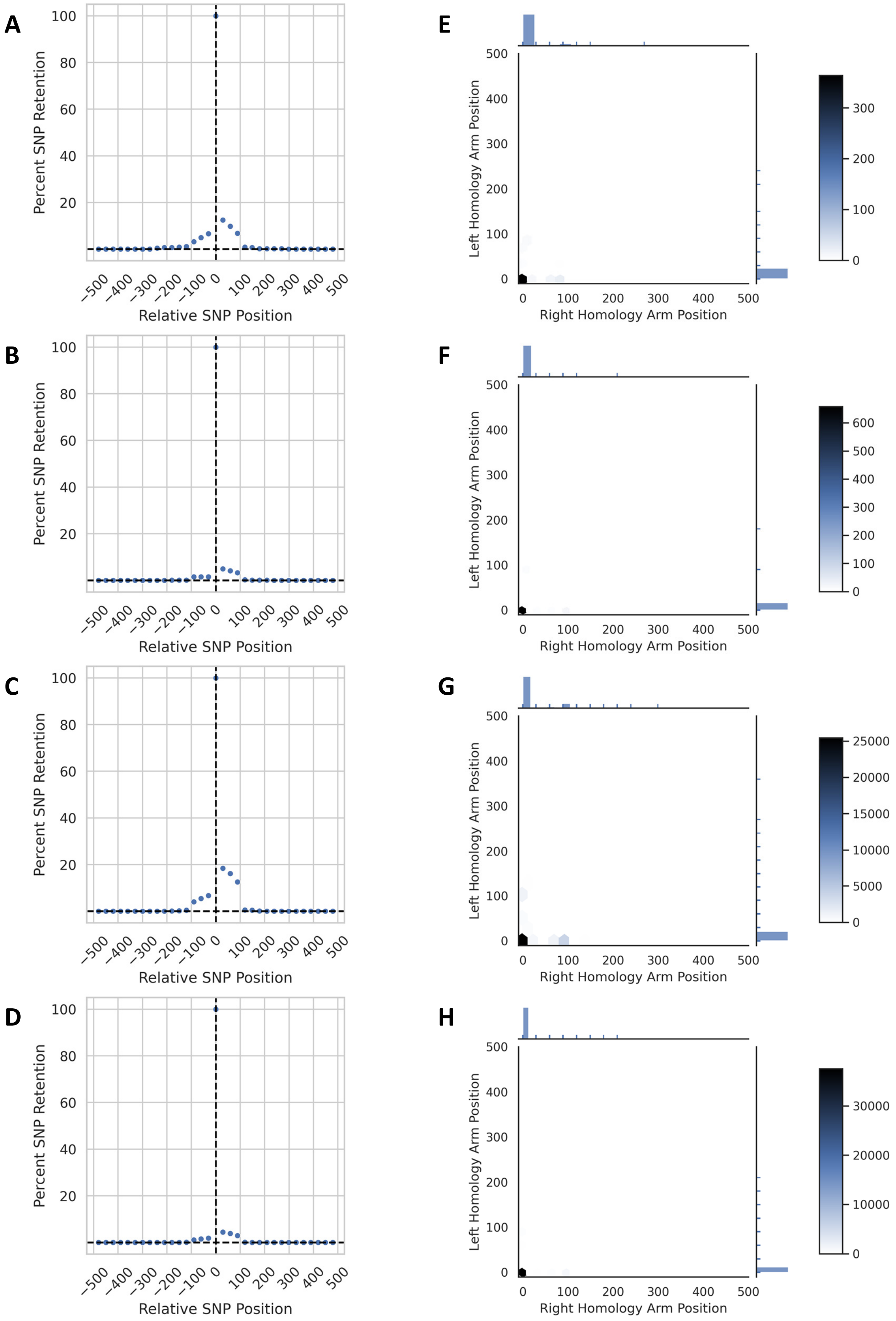
A
B
C
D
Percent SNP Retention
Relative SNP Position
100
80
60
40
20
−500
−400
−300
−200
−100
0
100
200
300
400
500
E
F
G
H
Left Homology Arm Position
Right Homology Arm Position
300
200
100
0
600
500
400
25000
20000
15000
10000
5000
30000

Figure S13. Negative control SNP conversion tracts at the *AGAMOUS locus.* SNP conversion tract background at the *AGAMOUS* target when delivered donors encoding 3bp insertions with perfect homology at gene targeting at non-gene targeting reads from representative samples **A)** Background conversion tract pattern from GT-positive reads when delivered a perfect homology donor encoding 3bp insertion and sgRNA. **B)** Background conversion tract pattern from GT-positive reads when delivered a perfect homology donor encoding a 3bp insertion, sgRNA, and GVR. **C)** As in A, but conversion tract patterns from non-GT reads. **D)** As in B, but conversion tract patterns from non-GT reads. **E, F, G, H)** As in A, B, C, and D, but visualized using a hexgrid, respectively.
